# Microscopic Observation Drug Susceptibility Assay (MODS) for Early Diagnosis of Tuberculosis in Children

**DOI:** 10.1371/journal.pone.0008341

**Published:** 2009-12-17

**Authors:** Dang Thi Minh Ha, Nguyen Thi Ngoc Lan, Marcel Wolbers, Tran Ngoc Duong, Nguyen Dang Quang, Tran Thi Van Thinh, Le Thi Hong Ngoc, Nguyen Thi Ngoc Anh, Tran Van Quyet, Nguyen Thi Bich Tuyen, Vo Thi Ha, Jeremy Day, Hoang Thi Thanh Hang, Vo Sy Kiet, Nguyen Thi Nho, Dai Viet Hoa, Nguyen Huy Dung, Nguyen Huu Lan, Jeremy Farrar, Maxine Caws

**Affiliations:** 1 TB Department, Pham Ngoc Thach Hospital, Ho Chi Minh City, Vietnam; 2 Wellcome Trust Major Overseas Programme and Oxford University Clinical Research Unit, Hospital for Tropical Diseases, Ho Chi Minh City, Vietnam; McGill University, Canada

## Abstract

MODS is a novel liquid culture based technique that has been shown to be effective and rapid for early diagnosis of tuberculosis (TB). We evaluated the MODS assay for diagnosis of TB in children in Viet Nam. 217 consecutive samples including sputum (n = 132), gastric fluid (n = 50), CSF (n = 32) and pleural fluid (n = 3) collected from 96 children with suspected TB, were tested by smear, MODS and MGIT. When test results were aggregated by patient, the sensitivity and specificity of smear, MGIT and MODS against “clinical diagnosis” (confirmed and probable groups) as the gold standard were 28.2% and 100%, 42.3% and 100%, 39.7% and 94.4%, respectively. The sensitivity of MGIT and MODS was not significantly different in this analysis (P = 0.5), but MGIT was more sensitive than MODS when analysed on the sample level using a marginal model (P = 0.03). The median time to detection of MODS and MGIT were 8 days and 13 days, respectively, and the time to detection was significantly shorter for MODS in samples where both tests were positive (P<0.001). An analysis of time-dependent sensitivity showed that the detection rates were significantly higher for MODS than for MGIT by day 7 or day 14 (P<0.001 and P = 0.04), respectively. MODS is a rapid and sensitive alternative method for the isolation of *M.tuberculosis* from children.

## Introduction

It is estimated that 9.27 million new tuberculosis (TB) cases occurred worldwide in 2000, of which approximately 11% were in children [1]. Importantly, 75% of these cases occur in 22 high-burden countries, including Viet Nam [1].

Pham Ngoc Thach Hospital in Ho Chi Minh City Viet Nam admits approximately 200 children with suspected TB each year. In 2007, only 14.9% (59/395) of treated pediatric TB cases had microbiological confirmation. The majority of confirmed cases had pulmonary TB. Confirmation of extrapulmonary TB is even more challenging in children. This is consistent with reports from other settings [2], [3].

The confirmation of TB is based on bacterial diagnosis. Sputum microscopy for TB diagnosis is widely used, rapid and straightforward but insensitive because of difficulty in obtaining adequate quality and volume of sputum samples from children. Lowenstein-Jensen (LJ) culture has been considered the gold standard for the diagnosis of TB for almost a hundred years, but it has a median time to positivity of four weeks and 70%–75% sensitivity against clinically defined probable adult cases. Commercial automated liquid culture methods including Mycobacterial Growth Indicator Tube (BATEC MGIT 960 - Becton Dickinson USA) are widely used in developed country settings for routine TB diagnosis. This method has a sensitivity of approximately 75% against clinically defined probable cases. Although such automated systems are faster than LJ culture, the median detection time is still approximately 14 days and the cost is relatively high, restricting their utility in high-burden settings[4]. Polymerase chain reaction (PCR) has not proved sensitive for the diagnosis of TB in children [5]. In 2007, there were approximately 60 suspected cases of tuberculous meningitis (TBM) in children admitted to Pham Ngoc Thach Hospital. However, only 3/60 (5%) of these cases had microbiological confirmation. Overall, the available techniques are time consuming, have low sensitivity or require expensive capital equipment or reagents. Therefore, it is necessary to develop new diagnostic methods that can compensate for the disadvantages of the established techniques.

Recently, a new method known as Microscopic Observation Drug Susceptibility Assay – MODS - has been evaluated for the diagnosis of TB in children at the Instituto de Salud del Niño of Lima, Peru [6]. It is a liquid culture based method which detects the living mycobacteria based on two well-known characteristics of M.*tuberculosis*; the growth in liquid medium is considerably faster than that on solid medium, and the distinctive morphology of M.*tuberculosis* in liquid culture as cording, strings or tangles. Recent evaluations have shown it to be an economical and rapid, sensitive and specific method for M.*tuberculosis* detection making it ideal for use globally but particularly in low income countries [7], [8]. Our previous study has shown that MODS can diagnose approximately 65% of all clinically diagnosed adult TBM in 6 days [9] with a higher sensitivity than smear (50%) and a faster turn around time than MGIT culture (12 days); making it ideal for determination of TB meningitis (TBM) in Viet Nam. The diagnosis of tuberculosis in children is frustrated by similar challenges as those experienced in adult TBM patients due to the paucibacillary samples and difficulty obtaining suitable sample volumes for testing. We assessed the effectiveness of MODS in diagnosis of tuberculosis in children.

## Methods

### Enrollment

All children ≤16 years of age with clinical suspicion of tuberculosis presenting to the paediatric ward at Pham Ngoc Thach Hospital, Ho Chi Minh City, Viet Nam from May 2008 to December 2008 were enrolled into the study. Any patient already receiving TB therapy for more than seven days was excluded from the study. Data on socioeconomic and demographic features, TB history, TB contact history, HIV status and presenting clinical features were prospectively collected on a standard case report form (CRF). Samples were collected as per routine care as deemed appropriate by the treating physician. No additional samples were collected as part of this study. All specimen types from suspected cases were included in the study except blood. The definition of TB was based on microbiological confirmation by either smear or MGIT, intention to treat, treatment management and outcome. Tuberculosis was defined as “confirmed TB” if the patient had clinical symptoms consistent with TB and either smear or MGIT was positive in any sample. A positive MODS was not considered as part of the ‘confirmed TB’ group definition because this was the test under evaluation.

The patient was defined as “probable TB” on ‘intention to treat’: if patients had clinical symptoms consistent with TB [10], had no microbiological confirmation by either smear or MGIT, but received TB treatment and were transferred to a District Tuberculosis Unit (DTU) for treatment and follow-up. Patients who satisfied the first two characteristics of “probable TB” but self-discharged with or without physician's permission were also classified in this group. It was impossible to either rule-out or confirm TB in this group due to the lack of microbiological confirmation.

Patients were defined as “TB unlikely” if they recovered without TB treatment, had TB treatment but deteriorated or received an alternative diagnosis and treatment. It was impossible to ‘rule-out’ TB in these patients because clinical deterioration may have been due to undetected drug-resistant TB although MDR rates are low in this population (<3%) [11]. HIV screening is not a routine test for pediatric patients at Pham Ngoc Thach Hospital. HIV counseling and testing was done if clinically suspected by the treating clinician, according to routine guidelines.

### Ethics

The protocol was approved by the Institutional Review Board (IRB) at Pham Ngoc Thach Hospital and the Health Services of Ho Chi Minh City. Informed consent was not sought because the study was conducted on routine samples only and it did not involve any intervention, additional samples or change in patient management. This patient consent waiver was approved by the IRB of Pham Ngoc Thach Hospital in the protocol.

#### Sample collection

All samples including sputum, cerebral spinal fluid (CSF), gastric fluid and pleural fluid were collected and transferred to the microbiology department on the same day (or the following day if they were collected after 4 pm). The samples were then submitted for smear, MGIT and MODS culture. The number of specimens per patient and the specimen type were decided by physicians.

#### Sample processing

All samples, except for CSF, were homogenised and decontaminated by Sputaprep (NaOH-NALC 2%) manufactured by Nam Khoa Company-Viet Nam prior to testing. The kit contains Mucoprep (NaOH 0.5 M and Na_3_Citrate 0.05, NALC (N-Acetyl-L-Cysteine) and Phosphate buffer (PO_4_ 10X - 0.67 M). Phosphate buffer 1X, homogenization buffer and decontamination buffer (HDB) were then prepared from the kit for sample processing. In brief, 3–5 ml sample was added to 3–5 ml HDB contained in a 50 ml falcon tube. The tube was shaken slightly by automated shaker and left at room temperature for 20minutes. After that, 35–39 ml phosphate buffer 1X was added into the mixture. The mixture was shaken by hand and then centrifuged at 3000 g, 4°C for 30 minutes. The supernatant was then discarded and 0.5 ml pellet at the bottom was re-suspended with 2 ml distilled water. The deposit was then aliquoted into 3 parts for smear, MGIT culture and MODS.

For CSF samples, the sample tubes were centrifuged at 3000 g, 4°C for 20 minutes (Eppendorf). After discarding the supernatant, 2 ml pellet was used for smear, MGIT and MODS culture. Technicians responsible for each test were blinded to the other microbiological results and clinical data on the patient records.

#### Homogenous smear

Two drops of pellet from each sample were put onto a slide for homogenous smear preparation. The smears were then stained by ZN method according to World Health Organisation (WHO) standard protocol [12].

#### MGIT culture

Processed samples were subjected to MGIT culture following the protocol of Becton Dickinson. In brief, 0.1 ml PANTA, 0.5 ml OADC and 0.5 ml of each processed sample were added into a MGIT medium tube. The mixture was inversion mixed by hand and then inoculated and incubated at 37°C in the MGIT machine. Positive results were reported automatically by the MGIT system.

#### MODS technique

The MODS culture was conducted in a biosafety cabinet class I which was placed in a separated room from the sample processing room, smear preparation room and MGIT culture room. The MODS method was performed as described in Park *et al.* [13] using the modification described by Caws et al. [9]. Briefly, MODS media was prepared with 5.9 g Middlebrook 7H9 broth (Difco, Sparks, MD), 3.1 ml glycerol and 1.25 g bacto casitone (Difco, USA) in 880 mls sterile distilled water. The media was autoclaved and stored in 22 ml aliquots at 4°C. Each new batch was tested for sterility by incubating one aliquot at 37°C for 1 week. Before use, OADC and PANTA (Becton Dickinson, USA) were added into each tube to final concentrations of 5.5% and 0.22% to make working MODS media. One 48-well MODS plate (Becton Dickinson, USA) was set up each day. Seven hundred and fifty mls of working MODS media was aliquoted to each well and 250 ml processed sample was added. One positive control (H37Rv) and one negative control well (sterile distilled water) were inoculated to each plate. Samples were inoculated into alternate wells to reduce cross-contamination. Empty wells contained MODS media. To prevent cross-contamination from evaporation, plate seals (optical flims, Biorad) were used. The plate was incubated at 37°C, and the result was recorded every alternate day after five days of inoculation for evidence of growth. Any cord formation including long cords or comma-shaped cords was recorded as positive and no cord was recorded as negative. Contamination was recorded if there was any growth or turbidity in any negative control well.

#### Subculture on LJ

All cultures positive by MODS or MGIT were subcultured on LJ media (Becton Dickinson) in duplicate and incubated at 37°C for several weeks. These isolates were then subjected to standard biochemical identification tests and DNA extracted and archived.

#### DNA extraction

DNA extraction was done using the CTAB method.[14] Briefly, mycobacteria were harvested from Lowenstein-Jensen slopes, placed into 400 ul of 1X TE, and incubated at 80°C for 20 min. After that, 50 ul of 10 mg/ml lysozyme, 75 ul of 10%SDS/proteinase K solution, 100 ul of 5 M NaCl and 100 ul of prewarmed CTAB/NaCl solution were added. The cells were incubated at 65°C for 10 min. DNA was extracted by the standard ethanol-chloroform extraction method.

#### Archiving

Isolates were archived in 7H9 media supplemented with 20% glycerol (7H9A). One or two loops of isolates collected from LJ culture were transferred into a cryotube containing 7H9A medium. These tubes were stored at −20°C.

#### Spoligotyping

Spoligotyping was done according to the standard international Spoligotyping protocol [15].

Spoligotyping was done for all cultures positive by MODS (n = 73). If MODS was contaminated while subculturing from MODS to LJ for DNA extraction (n = 2) or MODS was negative but MGIT positive (n = 8), cultures positive by MGIT were used for spoligotyping.

### Statistical Methods

Accuracy measures of the 3 tests were calculated for two different definitions of the ‘gold standard’ reference test: (1) microbiological confirmation (confirmed group) or (2) ‘clinical diagnosis’ (clinical gold standard including the probable and the confirmed group). In addition, we analyzed data on a ‘per patient’ or a ‘per sample’ basis.

For the ‘per patient’ analysis, the data was aggregated to provide one result per patient, i.e. the ‘per patient’ test was regarded as positive, if at least one sample yielded a positive test result. Reported confidence intervals for accuracy measures (sensitivities, specificities, positive and negative predictive values) were calculated according to the method of Pearson and Clopper. Comparisons of accuracies between tests were done using McNemar's test.

In the ‘per sample’ analysis we used a binary marginal generalized linear regression (GLM) models with an identity link function for all analyses. These models are very flexible, allow for the inclusion of covariates and account for the fact that results of multiple samples from the same patient or test results of different tests on the same sample may be dependent.[16] Specifically, we used marginal regression model to calculate confidence intervals for accuracy measures, to compare the sensitivities of smear, MGIT, and MODS and to assess the impact of the duration of TB treatment on the sensitivity of MODS.

For the ‘per sample’ analysis, we also calculated time-dependent sensitivity curves for MGIT and MODS. A test result was considered as positive by time *t* if the respective test was positive overall and reached the positive value at most *t* days after sample collection. Time-dependent sensitivity curves were estimated with the Kaplan-Meier method and samples without a positive test result were formally regarded as censored on day “infinity”. Time-dependent sensitivities of MGIT and MODS by days 7 and 14, respectively, were compared using a marginal regression model as described above. In addition, the time to positive MGIT and MODS, respectively, was compared in samples were both tests reached positivity with the Cox proportional hazards regression model. Robust standards were used to adjust for possible dependence of multiple samples from the same patient or test results of different tests on the same sample.

Comparison of demographic and clinical features of patients between TB diagnoses (definite, probable or unlikely) was done with Fisher's exact test for categorical data and the Kruskal-Wallis test for continuous data.

All reported confidence interval are two-sided 95% confidence intervals and p-values ≤0.05 were regarded as statistically significant. All analyses were done with Stata version 9 (Statacorp, Texas, USA) except graphs which were created with R version 2.90 (R Foundation for statistical computing, Vienna, Austria).

## Results

**Figure 1:**
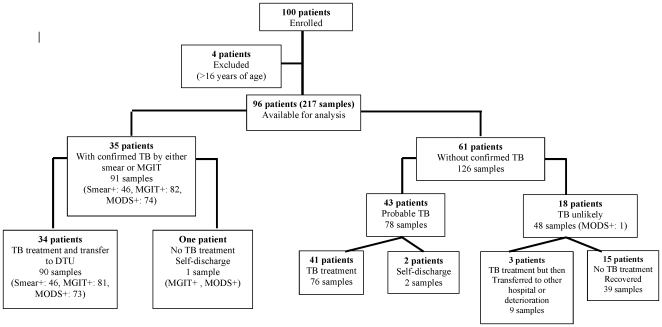
Patient recruitment and assignment of patients to ‘confirmed’, ‘probable’, or ‘TB unlikely’ groups.

One hundred children suspected of TB were enrolled into the study. Data of 96 (96%) children were included in the analysis. Four patients were excluded from the study because of not meeting the inclusion criteria (older than 16 years of age) (Figure 1). A total of 217 samples were collected: sputum (n = 132), gastric fluid (n = 50), CSF (n = 32), and pleural fluid (n = 3). Thirty five patients (36.5%, n = 35/96) had microbiological confirmation by a method other than MODS. 43 patients (44.8%, n = 43/96) were classified as ‘probable TB’ and 18 patients (18.8%, n = 18/96) were ‘TB unlikely’. There were three out of 96 patients who self-discharged before a final treatment decision was made. Samples were collected within 4 days after admission; and approximately 73% (70/96) patients provided samples in the first two days.

General demographic characteristics of the study population are shown in Table 1. In brief, the male: female ratio was 1∶1.3. The median age was 9 (IQR = 3–13). Almost 80% of suspects had evidence of BCG vaccination (neonatal BCG vaccination is routine in Viet Nam), only 5% had undergone previous TB treatment. Over 90% (n = 88/96) of suspects were not screened for HIV infection. Only patients suspected of HIV were counseled for HIV testing, under routine practice on the ward. Seven out of eight (87.5%) of those tested were positive. A quarter (24/96) of the study population had a TB contact according to parent interview and of those contacts 21/24 (87.5%) were a direct household member.

**Table 1:** Demographic characteristics of patients.

Characteristic	Total population N = 96	Confirmed TB N = 35	Probable TB N = 43	TB unlikely N = 18
**Gender**	P = 0.04	P1 = 0.002	P2 = 0.83	P3 = 0.50
Male	41 (42.7)	11 (31.4)	22 (51.2)	8 (44.4)
**Age**	P = 0.005	P1 = 0.004	P2 = 0.34	P3 = 0.07
(year) Median (IQR)	9 (3–13)	13 (5–14)	6(2–11)	8.5 (4–12)
BCG vaccination^(*)^	P = 0.31			
Yes	74 (77.1)	27 (77.1)	32 (74.4)	15 (83.3)
No	21 (21.9)	8 (22.9)	11 (25.6)	2 (11.1)
Unknown	1 (1.0)	0	0	1 (5.56)
**TB treatment history**	P = 0.86			
Yes	5 (5.2)	2 (5.7)	2 (4.7)	1 (5.6)
No	89 (92.7)	33 (94.3)	39 (90.70)	17 (94.4)
Unknown	2 (2.1)	0	2 (4.6)	0
**HIV status**	P = 0.34			
Positive	7 (7.3)	2 (5.7)	3 (7.0)	2 (11.1)
Negative	1 (1.0)	0	0	1 (5.6)
Unknown	88 (91.7)	33 (94.3)	40 (93.0)	15 (8.3)
**ARV therapy** (only for HIV positive)	P = 0.85			
Yes	2 (28.6)	0	2 (66.7)	0
No	5 (71.4)	2 (100)	1 (33.3)	2 (100)
**TB contact** (Parent report)	P = 1.0			
Yes	24 (25.00)	9 (25.7)	11 (25.6)	4 (22.2)
Contact family member	21/24 (87.5)	7/9 (77.7)	10/11(90.9)	4/4 (100)
No	72 (75.00)	26 (74.3)	32 (74.4)	14 (77.8)

Summary measure is n (%) for all categorical characteristics.

(*). Scar or parent report.

P value for comparison of all three groups. If P<0.05, P1, P2, P3 will be calculated.

P1: for comparison between confirmed TB and probable TB; P2: for between probable TB and TB unlikely and P3: for between TB unlikely and confirmed TB.

Clinical symptoms of TB of this referral study population included cough (67.7%), fever (80.2%), weight loss (45.8%) and chest X-ray consistent with TB (75%) (Table 2). Other symptoms were haemoptysis, convulsion, unconsciousness, difficulty breathing and vomiting. The median history of illness was 20 days. The ‘confirmed TB’ patient group reported cough more frequently than ‘probable’ TB patients (P = 0.03) and had a chest X-ray consistent with TB more often (P = 0.005) than probable TB patients.

**Table 2:** Clinical features of 96 pediatric TB suspects.

Characteristic	Total population N = 96	Confirmed TB N = 35	Probable TB N = 43	TB unlikely N = 18
**History of illness**	P = 0.89			
Median days (IQR)	20 (14–30)	15 (12–30)	20 (14–30)	24.5 (10–30)
**Cough**	P = 0.02565 (67.7)	P1 = 0.0327 (77.1)	P2 = 0.0323 (53.5)	P3 = 0.5915 (83.3)
**Fever**	P = 0.3677 (80.2)	27 (77.1)	37 (86.1)	13 (72.2)
Nightsweat	P = 0.6333 (34.4)	11 (31.4)	17 (39.5)	5 (27.8)
**Weightloss**	P = 0.3744 (45.8)	15 (42.9)	18 (41.9)	11 (61.1)
**Failure to thrive**	P = 0.4818 (18.7)	5 (14.3)	8 (18.6)	5 (27.8)
**Lymphadenopathy**	P = 0.3814 (14.6)	7 (20.0)	4 (9.3)	3 (16.7)
**Chest X-ray suspected of TB**	P = 0.025	P1 = 0.005	P2 = 0.08	P3 = 0.59
Yes	72 (75.0)	31 (88.6)	26 (60.5)	15 (83.3)
No	20 (20.8)	3 (8.6)	15 (34.9)	2 (11.1)
Unknown	4 (4.2)	1 (2.9)	2 (4.6)	1 (5.6)
**Radiological description** (for chest X-ray suspected of TB only)				
Cavity	13 (18.1)	10 (32.3)	1 (3.9)	2 (13.3)
Miliary	3 (4.2)	1 (3.2)	1 (3.9)	1 (6.7)
Infiltrate	28 (38.9)	7 (22.6)	14 (53.9)	7 (46.7)
Nodular lesion	9 (12.5)	5 (16.1)	2 (7.7)	2 (13.3)
Instertitial infiltration	1 (1.4)	1 (3.2)	0	0
Shadowing	12 (16.7)	5 (16.1)	4 (15.4)	3 (20.0)
Hillar enlargement	4 (5.6)	2 (6.5)	2 (7.7)	0
No description	2 (2.8)	0	2 (7.7)	0

Summary measure is n (%) for all categorical characteristics.

P value for comparison of all three groups. If P<0.05, P1, P2, P3 will be calculated.

P1: for comparison between confirmed TB and probable TB; P2: for between probable TB and TB unlikely and P3: for between TB unlikely and confirmed TB.

### Accuracy of MODS

#### Clinically diagnosed TB as the gold standard

The clinically diagnosed gold standard was defined as patients who satisfied characteristics of either “confirmed TB” group or “probable TB” group. 78 patients were diagnosed with TB by clinical features and/or microbiological confirmation and 18 patients were classified as TB unlikely.

When analysed by patient, the sensitivity of smear, MGIT and MODS were 28.2%, [95%CI: 18.6, 39.5] (n = 22/78), 42.3%, [95%CI: 31.1, 54.0] (n = 33/78) and 39.7%, [95%CI: 28.8, 51.4] (n = 31/78). MODS was more sensitive than smear (P = 0.011) [95%CI of difference: 2.3%, 20.7%] but comparable to MGIT (P = 0.50; 95%CI of difference [−2.2%, 7.3%]). Specificity, positive predictive value (PPV) of smear and MGIT was 100% because it was part of the definition for confirmed TB, but all patients with a positive smear/MGIT had clinical symptoms consistent with TB and were considered to have TB by the treating clinician. Specificity and PPV of MODS were 94.4%, [95%CI: 72.7, 99.8] (n = 17/18) and 96.8%, [95%CI: 83.7, 99.9], (n = 31/32), respectively. Negative predictive value (NPV) of smear, MGIT and MODS was 24.3%, [95%CI: 15.0, 35.6] (n = 18/74]; 28.6%, [95%CI: 17.8, 41.3] (n = 18/63) and 26.6%, [95%CI: 16.2, 39.0] (n = 17/64).

When analysed by sample, the sensitivity of MODS, MGIT and smear was 43.8% [95%CI: 32.8, 54.7] (n = 74/169), 48.5% [95%CI: 36.9, 60.0] (n = 82/169) and 27.2% [95%CI: 17.0, 37.4] (n = 46/169), respectively. MODS was more sensitive than smear (P<0.001; 95% CI for difference [8.5%, 24.6%]) but less sensitive than MGIT (P = 0.027; 95%CI of difference [−8.9%, −0.5%]). Table 3 shows the sensitivity of the three methods against clinical diagnosis as the gold standard in terms of patient and sample.

**Table 3:** Sensitivity of MODS, smear and MGIT against clinical gold standard.

		MODS n(%) [95%CI]	SMEAR n(%) [95%CI]	MGIT n(%) [95%CI]	Comparison: P-value, [95%CI of difference]	
					MODS vs SMEAR	MODS vs MGIT
**By patient** (N = 78)		31 (39.7) [28.8, 51.4]	22 (28.2) [18.6, 39.5]	33 (42.3) [31.1, 54.0]	0.011, [2.3%, 20.7%]	0.5, [−2.2%, 7.3%
**By sample** (N = 169)		74 (43.8) [32.8, 54.7]	46 (27.2) [17.0, 37.4]	82 (48.5) [36.9, 60.0]	<0.001, [−2.5%, −8.5%]	0.027, [−8.9%, −0.5%]
**By Sampletype**	Sputum (N = 101)	63 (62.4) [48.1, 76.5]	42 (41.6) [26.5, 56.6]	68 (67.3) [52.8, 81.8]	<0.001, [9.6%, 31.9%]	0.051, [−9.9%, 0.03%]
	Gastric fluid (N = 35)	10 (28.6) [5.1, 52.1]	3 (8.6) [0.0, 20.5]	10 (28.6) [6.1, 51.0]	0.045,[−39.5%,−0.4%]	1, [−8.2%, 8.2%]

#### Microbiological confirmation as the gold standard

**Figure 2:**
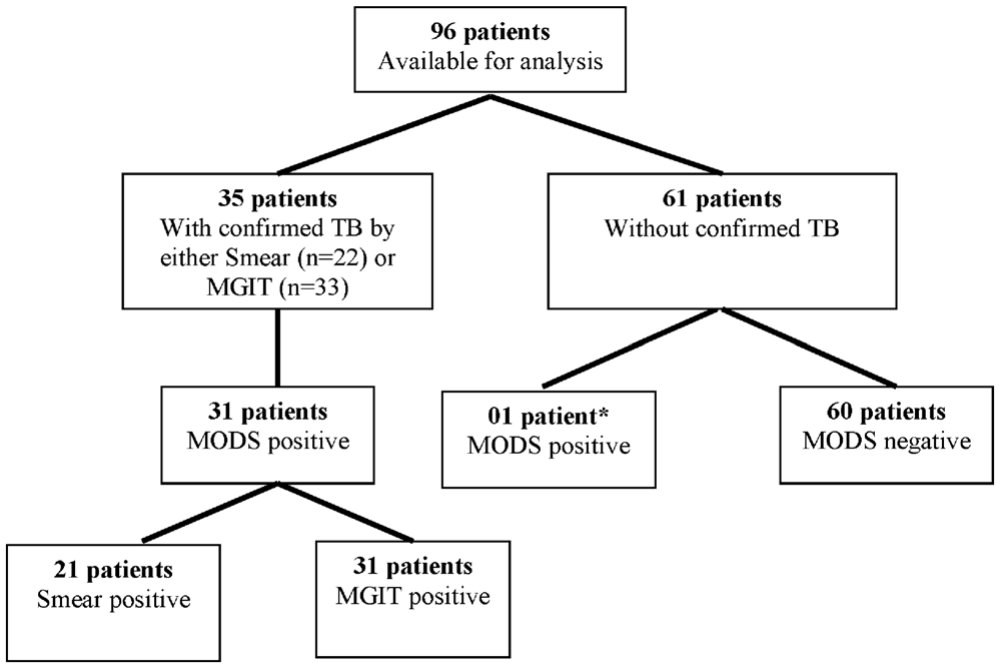
MODS positive in relation to Smear and MGIT, by patient. * This patient was deemed a false positive due to H37Rv (the positive control strain) identification by spoligotyping.

Of 35 patients with confirmed TB, 31 (88.6%) patients had at least one sample positive by MODS (*Figure 2*). When analysed by patient, the sensitivity, specificity, PPV and NPV of MODS were 88.6% [95%CI: 73.0, 96.0] (n = 31/35), 98.4% [95%CI: 91.0, 99.0] (60/61), 96.88% [95%CI: 83.0, 99.0] (n = 31/32) and 93.8% [95%CI: 84.0, 98.0] (n = 60/64), respectively against microbiological confirmation. The specificity of MODS was not 100% because there was one patient positive by MODS but negative by both smear and MGIT. Spoligotyping of the MODS positive isolate of this patient, showed it to be H37Rv, which was used as the positive control in the MODS technique and this result was therefore classified as false positive by cross-contamination. Clinical diagnosis in this patient was microbiologically confirmed Staphylococcus *aureus* infection.

When analysed by sample, MODS yielded a sensitivity of 81.3% [95%CI: 73.3, 89.2] (n = 74/91), specificity of 99.2% [95%CI: 97.7, 100] (n = 125/126), PPV of 98.6% [95%CI: 96.0, 100] (n = 74/75) and NPV of 88.0% [95%CI: 81.7, 94.4] (n = 125/142). Using MODS we recultured 8 of the samples which had been positive by MGIT but negative by MODS. These samples remained culture negative by the MODS technique.

### Effect of Sample Type

To investigate whether sample type had a strong impact on the sensitivity of the three methods, we investigated the number of each sample type collected from 78 clinically diagnosed TB patients and analyzed the sensitivity of these methods in terms of sputum sample (n = 101), gastric fluid (n = 35), CSF (n = 30) and pleural fluid (n = 1) (table 3). Our data showed that in sputum, the sensitivity of MODS (62.4%, n = 63/101) was significantly higher than smear (41.6%, n = 42/101), P<0.001; 95% CI for difference [9.6%, 31.9%]; and marginally lower than MGIT (67.3%, n = 68/101) with P = 0.051; 95%CI of difference [−9.92%, 0.03%]. In gastric fluid, MODS and MGIT had the same sensitivity (28.6%, n = 10/35); and they were more sensitive than smear (P = 0.045; 95% CI [0.4%, 39.5%]). In CSF samples, no positive result was detected by smear. MODS detected one CSF and MGIT was positive with 4 CSF samples. There were insufficient samples to analyze the sensitivity of these methods in CSF (n = 30) and pleural fluid (n = 1).

### Effect of Treatment

**Figure 3:**
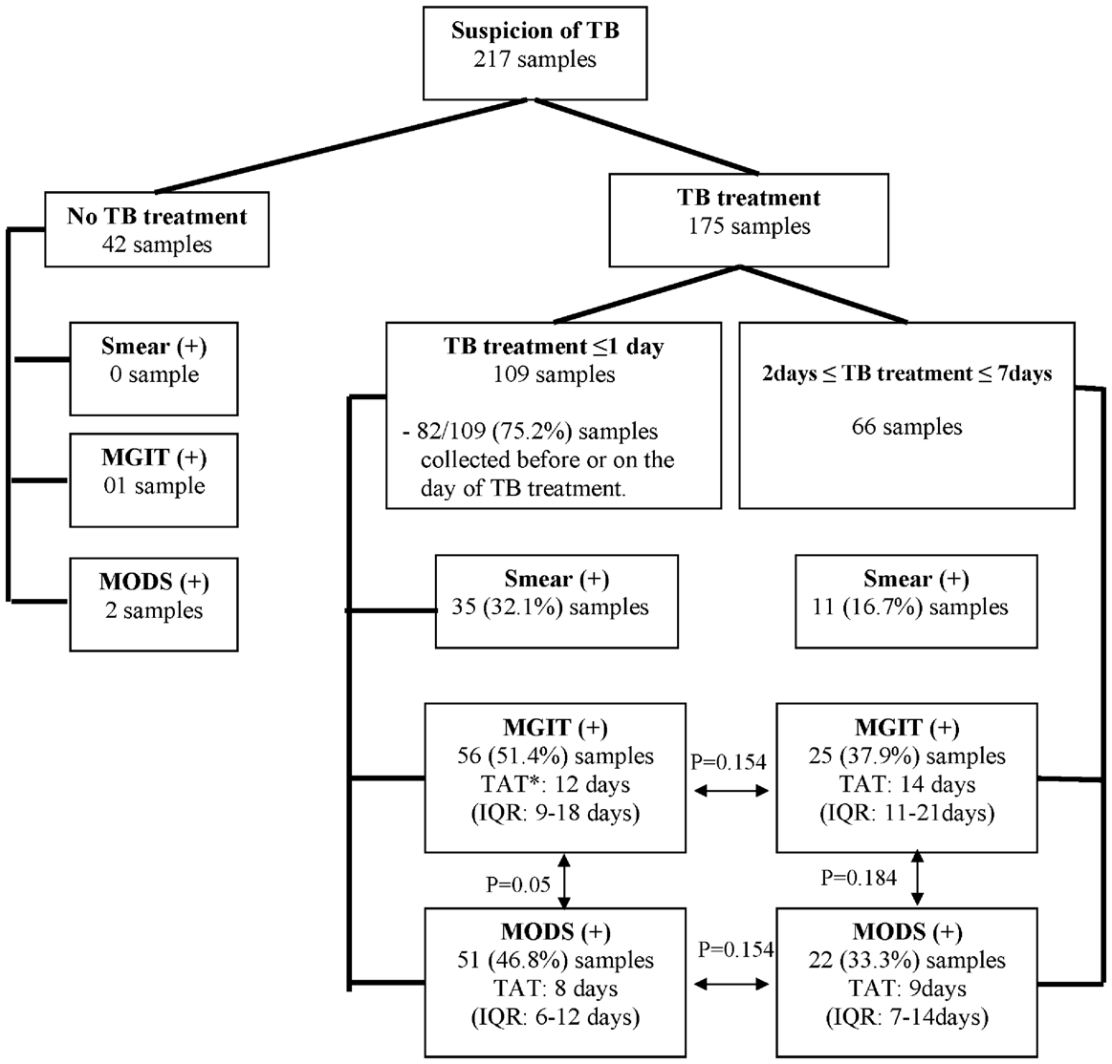
Detection rates of Smear, MGIT and MODS in relation to TB treatment. P values for comparison of detection rates between MODS and MGIT. * TAT: Turn around time.

175 samples of all types from patients receiving TB treatment in this admission were collected on the day of TB treatment or within seven days of starting TB therapy. TB treatment for more than 1 day reduced the sensitivity of all 3 microbiological tests, but this difference did not reach significance; (P = 0.054; 95%CI of difference [−31.1%–0.2%]) for smear, (P = 0.154; 95%CI [−32.1%, 5.0%]) for MGIT and (P = 0.154; 95%CI [−31.9%, 5.0%]) for MODS (figure 3).

### Time to Detection

Time to detection was defined as the number of days from sample processing (day 1) to results available. Smear results were available one day after sample processing due to the high volume of samples in this laboratory. Median time to detection of MODS was faster than MGIT, 8 days (IQR: 6–12) vs 13 days (IQR: 9–19), respectively in samples positive by MODS or MGIT. In the 73 samples which were both MGIT and MODS positive, MODS was faster in 64 (88%) samples and the median (IQR) time difference was 3 (1 to 6) days in favor of MODS (p<0.001).

**Figure 4:**
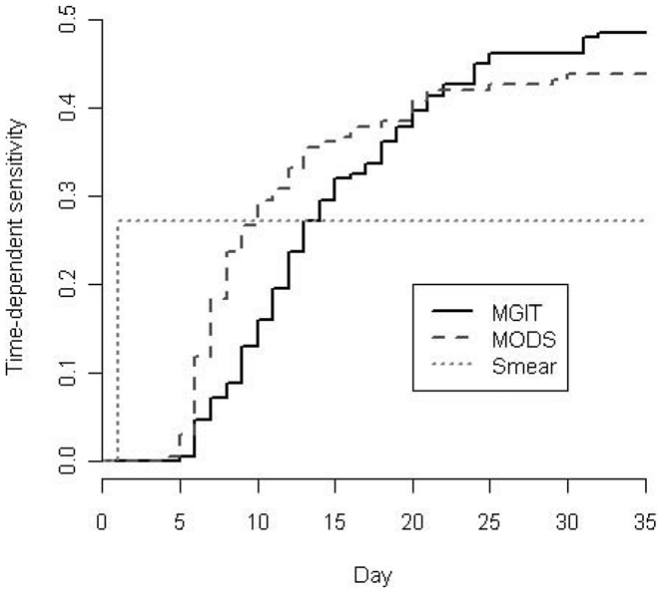
Time-dependent sensitivity of smear, MODS and MGIT. In the 73 samples which were both MGIT and MODS positive, the time dependent sensitivities of MODS were higher than MGIT on both day 7 (P<0.001) and day 14 (P = 0.04).

“Time-dependent sensitivity” counted only samples positive by both MGIT and MODS which reached positivity by day 7 or 14, respectively. The time-dependent sensitivity against clinical gold standard was significantly higher for MODS compared to MGIT on both day 7 (18.34% vs. 7.10% P = <0.001), and on day 14 (36.09% vs. 29.59%, P = 0.04) (figure 4).

Four CSF samples positive by MGIT had a median time to positive of 24.5 days (IQR: 23–25). One CSF sample was positive by MODS 6 days after inoculation.

### Detection of Smear Negative TB

To evaluate if MODS is useful for detection of smear negative cases, we evaluated the sensitivity of MGIT and MODS among 56 smear negative TB cases (confirmed and probable) and 123 smear negative TB samples including either MODS or MGIT positive. The sensitivity of MGIT and MODS by patient were comparable, (P = 0.5; 95%CI for difference [−3.1%, 10.2%]), which were 21.4% (n = 12/56) and 17.85 (n = 10/56), respectively. The sensitivities were 30.08%, [95%CI: 18.5, 41.6] (n = 37/123) and 25.20% [95%CI: 14.9, 35.5](n = 31/123) for MGIT and MODS, respectively, in terms of sample with P = 0.053, 95%CI for difference [−0.05%, 9.80%]. The median time to positive of MODS for smear negative samples was considerably faster than MGIT, which was 12 days (IQR: 8–17 days) compared to 19 days (IQR: 15–22 days). In the 30 samples where both MGIT and MODS were positive, the median time difference was 6 days (IQR: 3–11days) in favor of MODS (p = 0.005). Therefore, MODS appeared to have an advantage over MGIT in terms of time to detection.

### Time to Positive in Relation to Smear Result

**Figure 5:**
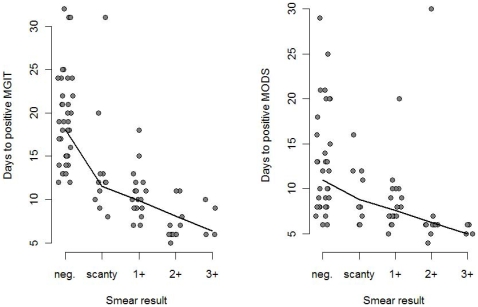
Time to MGIT positive and MODS positive in relation to smear grade. Filled dots are samples positive by either MGIT or MODS, lines are scatter plot smoothers. Both MGIT and MODS had a negative Spearman rank correlation with smear grade (P<0.001).

To investigate whether time to positive of MGIT and MODS is related to smear positive grading, we analyzed the association of time to detection of MGIT and MODS with smear grade. Figure 5 demonstrates a negative correlation of the time to a positive test for both MGIT and MODS, respectively, compared to smear with both P = <0.001.

### Cerebral Spinal Fluid

One CSF was collected from each patient with suspected TBM. Thirty-two out of ninety-six patients (33.4%) had a lumbar puncture performed to establish a diagnosis of TBM. Of these, 6 patients were classified as confirmed TBM (The detection results are as following: 3 patients had culture positive CSF by MGIT only, one patient had CSF positive by both MODS and MGIT, one patient had positive sputum by all three methods and one patient had smear positive gastric fluid), 24 patients were defined as probable TBM on ‘intention to treat’ basis as previously published criteria [17] and 2 patients were defined as ‘TBM unlikely’. In brief, CSF appearance was usually clear (68.8%), the median CSF glucose/blood glucose ratio was 0.33, median CSF protein was 0.46 g/l (IQR: 0.26–0.63), median cell count was 47 cells/mm^3^ (IQR: 13–110) and 100% were lymphocytic (IQR: 100%–100%). CSF parameters were not significantly different among groups (data not shown). The CSF volume available for testing varied with a median value of 3 ml (IQR: 2.5 ml–5 ml). The median CSF volume of children in the younger age group (under five years of age) (n = 19) was 3 ml (IQR: 2 ml–3 ml) and it was 5 ml (IQR: 5 ml–5 ml) in the older group (over 12 years old) (n = 4).

### Contamination and Spoligotyping

In terms of contamination with fungi, the original contamination rate of MGIT and MODS were 1.39% (n = 3/217) and 0.9% (n = 2/217), respectively. All MGIT contaminated samples were decontaminated again and reinoculated in MGIT medium. No contamination was observed after decontamination. Reprocessing for sample contaminated by MODS was not attempted because of low volume (total of 1 ml for each well).

**Figure 6:**
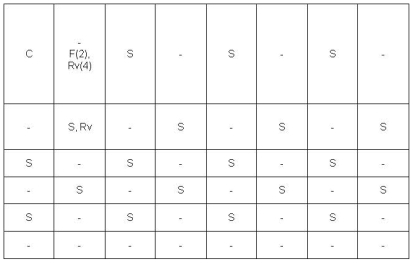
Position of contamination observed in MODS Plates. C. Positive control (H_37_Rv), S. Sample, F. Contaminated with fungi, Rv. Contaminated with H37Rv, Rv(4). Four experiments contaminated with H37Rv at this position.

In terms of cross-contamination with TB bacteria, one sample and four negative control wells were contaminated with positive control isolate (H_37_Rv) at the wells closest to the positive control well. Figure 6 shows the positions of contamination observed in the MODS experiment. The average number of samples processed on each day having contamination was 8, ranging from 3 samples to 21 samples.

One isolate that was culture positive by MGIT but negative by MODS was not transferred for genotyping because of contamination during archiving. Serial isolates or isolates from different samples from the same patient had identical spoligotypes except in 2 cases. One patient had different genotypes isolated from MGIT and MODS cultures. Spoligotyping was used to investigate the possibility of cross-contamination of the MODS culture. No other samples with the same spoligotype were processed for MODS on that day, therefore this may have been a cross-contamination in MGIT culture or a patient with dual infection. The patient had symptoms consistent with TB and was considered to have TB by the treating clinician and received TB treatment.

In another patient, a single MGIT culture was contaminated with *Mycobacterium fortuitum* on subculture. However, 3 MODS positive cultures and 2 MGIT cultures from this patient isolated *M.tuberculosis* with identical genotypes.

## Discussion

This study demonstrates that MODS is a suitable, rapid, sensitive and specific test for the diagnosis of paediatric TB.

With clinical diagnosis as the gold standard, MODS was more sensitive than smear (P = 0.011) and as sensitive as MGIT (P = 0.5) on per patient analysis (n = 78),. However, on per sample analysis (n = 169), MODS was more sensitive than smear (P<0.001) but less sensitive than MGIT (P = 0.027). Larger sample size (169 samples compared to 78 patients) may account for this difference.

With microbiological diagnosis, MODS diagnosed 88% of TB cases, similar to a report from Peru in 2006 in which MODS detected 87% of pediatric TB cases [6]. Although sensitivity of MODS was not statistically different from MGIT in terms of patient analysis, MGIT detected marginally more TB patients than MODS (33/35 patients vs 31/33 patients). One explanation for this difference may be the volume of sample inoculated in each well. It is known that sample volume is important in TB diagnosis for paucibacillary samples; an increased volume increases the likelihood of bacilli in the sample [18].We applied 0.5 ml of decontaminated sample for MGIT culture while only 0.25 ml (half volume) was used for MODS. In another study in adults, 0.72 ml of decontaminated sputum was used for MODS (almost three times higher than our volume) and 0.5 ml was used for MBBacT [7]. In that study, the sensitivity of MODS was higher than MBBacT (97.8% vs 89.0%). In our laboratory, 48 well-plates are used instead of 24 well-plates, which allows for faster plate reading, an important consideration in high-burden laboratories however this lowers the deposit volume inoculated which may have an impact on sensitivity.

Pediatric TB samples are paucibacillary and it is therefore important to consider which sample types yield highest sensitivity from the limited sample volumes that are inevitably available from children. Our study found that sputum and gastric fluid were appropriate for use to diagnose pediatric TB, in agreement with other groups who have also found gastric fluid to improve confirmed TB rates in sputum-negative pediatric TB. [6].

In patients where both tests MODS and MGIT reached positivity, the time to positive of MODS was shorter than MGIT with a median time difference of 3 days to 6 days. Technical issues affect the turn around time of each method. In MGIT culture, 10^5^ to 10^6^ bacteria/ml are required for the machine to signal a positive result. In MODS culture, the result was read under an inverse microscope with 400× magnification. Therefore, a few bacteria in each well can be detected by MODS several days before MGIT. From this study, we also observed that the time to positive is inversely related to the number of bacilli in each sample or smear results. Since the sensitivity of MODS and MGIT were similar in smear negative samples and MODS has a faster time to positive than MGIT, MODS is a candidate method for routine use to diagnose smear negative pediatric TB.

The specificity of MODS (98.4%) was slightly lower than smear (100%) and MGIT (100%) because we had one false positive sample due to cross-contamination with H_37_Rv. Our results were similar to the results of a study conducted in 2006 which recorded a specificity of 99.6% due to cross-contamination between samples. [7]. All positive samples in this present study were identified as *M.tuberculosis* by spoligotying and no sample with cross-sample contamination was detected. No mis-identification of Non-tuberculous mycobacteria (NTM) was recorded in this study but this needs further evaluation in populations with higher rates of NTM, such as patients coinfected with HIV.

Contamination is a common problem with all liquid culture and MODS is not an exception; careful manipulation and rigorous aseptic technique must be maintained to minimize contamination. Contamination with fungi was rare at only 1.4%. We have not detected any cross-contamination between samples but we did have cross-contamination from the positive control isolate (H_37_Rv) to a single sample and to negative control wells; all cross-contaminated wells were sited at the positions closest to the positive control wells. A high concentration of H_37_Rv is inoculated into positive control wells (>10^5^ bacteria/100 ul), and therefore cross-contamination was more likely from the positive control than from paucibacillary samples.

The MODS technique requires a centrifuge, class I cabinet, standard NALC/NaOH decontamination solution, MODS media (7H9 supplemented with OADC, glycerol and PANTA), inverted microscope and tissue culture plates. In addition, the cost of MODS is low (approximately 1.1USD per sample, excluding labour and capital equipment) and so, MODS should be evaluated for application in provincial TB laboratories within national TB programs.

Overall, MODS is a sensitive, quick and reliable culture test for use in the diagnosis of children with TB, allowing these vulnerable individuals to get onto TB treatment earlier. It is also cheaper than the alternative rapid culture or molecular methods currently available. Further studies should be done to evaluate the specificity, cross-contamination, and feasibility of MODS in National TB Programs.
